# Survival-Associated Alternative Splicing Events and Prognostic Signatures in Pancreatic Cancer

**DOI:** 10.3389/fgene.2020.522383

**Published:** 2020-09-30

**Authors:** Lichao Xu, Jingxin Pan, Yanni Ding, Hongda Pan

**Affiliations:** ^1^Department of Interventional Radiology, Fudan University Shanghai Cancer Center, Shanghai, China; ^2^Department of Oncology, Shanghai Medical College, Fudan University, Shanghai, China; ^3^Department of Internal Medicine, The Second Affiliated Hospital of Fujian Medical University, Quanzhou, China; ^4^Department of Surgery, Shaan Xi Provincial Tumor Hospital, Xi’an City, China

**Keywords:** pancreatic cancer, alternative splicing, splicing factors, prognosis, The Cancer Genome Atlas

## Abstract

**Background:**

Alternative splicing (AS) is reported to be related to the biological process of multiple malignancies. This study is conducted to identify survival-associated AS events and prognostic signatures that may serve as prognostic indicators for pancreatic cancer (PC).

**Methods:**

Univariate Cox analysis was used to determine the survival-associated AS events in PC. Prognostic signatures were constructed by LASSO Cox analysis based on seven types of survival-associated AS events. The correlation between the expression of splicing factors (SFs) and the percent spliced in values of AS events was analyzed by Pearson correlation analysis. Risk scores were calculated to determine high- or low-risk patients with different types of AS events. Gene functional annotation analysis was performed to reveal pathways in which prognostic AS is enriched.

**Results:**

A total of 45,313 AS events in 10,624 genes were observed, and there were 1,565 AS events in 1,223 genes significantly correlated with overall survival for PC. Kaplan–Meier analysis, receiver-operator characteristic curve, univariate and multivariate Cox analyses showed that AS prognostic signatures could effectively predict prognosis of patients with PC. Splicing factors–AS regulatory networks were established to demonstrate the interaction between AS events and SFs.

**Conclusion:**

The survival-associated AS events and prognostic signatures identified in this study can serve as useful tool for predicting prognosis of patients with PC. Moreover, the SF–AS regulatory networks may provide clues for the mechanisms underlying AS in PC.

## Introduction

Alternative splicing (AS) is a crucial posttranscriptional biological process that is responsible for the modification of mRNA isoforms. By facilitating transcript variants and reprogramming of protein diversity in cells, AS plays an important role in generating various mRNA and protein isoforms (Yang et al., 2016; Climente-González et al., 2017). There are seven types of AS events, namely alternate acceptor site (AA), alternate donor site (AD), alternate promoter (AP), alternate terminator (AT), exon skip (ES), mutually exclusive exon (ME), and retained intron (RI). Accumulating evidence has suggested that abnormal AS is associated with the aberrant cellular homeostasis and oncogenic processes of multiple malignancies (Jin et al., 2018; Wong et al., 2018). Investigating mechanisms underlying AS deepens our understanding of posttranscriptional regulatory patterns.

Substantial progress in high-throughput sequencing technology has greatly promoted research at a whole-genome scale. RNA sequencing data generated by The Cancer Genome Atlas (TCGA) program facilitated the in-depth research to illustrate the profiling of AS. By collecting and analyzing RNA sequencing data from TCGA database, SpliceSeq (Ryan et al., 2016) provides processed data for the analysis of AS events in 33 types of cancers (Lin et al., 2018; Zhong et al., 2018; He et al., 2019). Recently, researchers have found the clinical significance of AS, and it may serve as a prognostic predictor for several types of cancers (Singh and Eyras, 2017; Huang et al., 2018; Zhong et al., 2018; Chen et al., 2019; Mao et al., 2019; Zhu et al., 2019). However, a comprehensive study regarding aberrant AS events in pancreatic cancer (PC) is lacking.

Pancreatic cancer is one of the most common malignant tumors of the digestive system due to its latent onset, difficulty in surgical resection, poor prognosis and high mortality rate (Bray et al., 2018). Surgical removal constitutes one of the most common and effective treatments for PC and is often the only curative treatment option (Neoptolemos et al., 2018). Histopathological criteria do not adequately inform treatment decisions for PC. Therefore, it is of great importance to develop novel prognostic biomarkers to accelerate therapeutic development of PC.

In the present study, we investigated the profiles of aberrant AS events and its clinical and prognostic implications in patients with PC. Survival-associated AS events were identified, and AS prognostic signatures were constructed to predict the prognosis of PC. Furthermore, a regulatory network was also established to determine the interaction among splicing factors (SFs) and AS in PC.

## Materials and Methods

### AS Data Acquisition and Process

Clinical information of 177 patients with PC was also downloaded and extracted from TCGA database. The overall survival (OS) was used as the endpoint for survival. A total of 173 patients were enrolled in the subsequent analyses after the survival data integrated with AS data, four patients were excluded for lack of AS data. The percent spliced in (PSI) value was processed by TCGA SpliceSeq to quantify AS events. The PSI value indicates the inclusion of a transcript element divided by the total number of reads for that AS event. Alterations in PSI values range from 0 to 1, which suggests a shift percentage in splicing events. Alternative splicing events with percentage of samples with PSI value of more than 75% in a pancreatic adenocarcinoma (PAAD) cohort were obtained from TCGA SpliceSeq website. UpSet plots created by the package “UpSetR” (Conway et al., 2017) in R software was used to analyze and demonstrate the intersection and distribution among seven types of AS.

### Survival Analysis

According to the survival data from TCGA, the follow-up periods of PC patients ranged from 31 to 2182 days after excluding patients with an OS of less than 30 days. Univariate Cox analysis was used to identify survival-associated AS events by analyzing the correlation between the survival status of patients with PC and PSI value (from 0 to 1) of each AS event (*P* < 0.05).

### Construction and Evaluation of Prognostic Signature

The top 20 most significant events of seven types of AS identified from the univariate Cox analysis were submitted to a least absolute shrinkage and selection operator (LASSO) analysis to construct prognostic signatures. The “glmnet” package (Engebretsen and Bohlin, 2019) in R was used to calculate the coefficients and partial likelihood deviance of the signatures. The risk score for OS prediction were calculated by multiplying the coefficients assigned by LASSO Cox analysis and the PSI values of AS events. Multivariable Cox regression analyses were conducted to evaluate the role of AS prognostic signatures as independent predictors. Time-dependent receiver-operator characteristic (ROC) curves were generated to determine the prognostic prediction efficacy of the AS signatures using the package “survivalROC” (Heagerty et al., 2000) in R software. The Kaplan-Meier survival analysis was performed to assess the survival difference between low- and high-risk groups.

### Construction of a SF-AS Regulatory Network

A list of 404 SFs that has been previously reported was obtained from SpliceAid 2 database (Piva et al., 2012). The expression profiles of SF genes were selected from TCGA dataset. Pearson’s correlation analysis was conducted to assess the correlation between the PSI values of prognostic AS events and the expression of SFs. Splicing factors–AS interactomes with *P* value less than 0.05 and a correlation coefficient more than 0.6 were enrolled to construct the SF–AS a regulatory network via Cytoscape version 3.6.1.

### Gene Ontology Analysis

Functional annotation of genes with prognostic AS events was conducted with the package “clusterProfiler” (Yu et al., 2012) in R. Biological Process (BP) of Gene ontology (GO) were used to assess the functional categories. Gene ontology-BP terms with a *P*-value less than 0.05 were considered significant categories.

## Results

### Profiles of Alternative Splicing Events in PC

**FIGURE 1:**
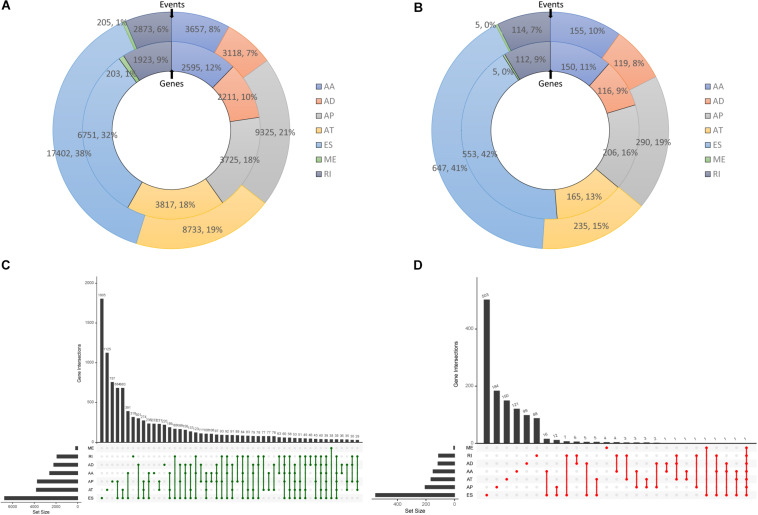
Overview of alternative splicing (AS) events and survival-associated AS events in PC. **(A)** Numbers and percentages of events and corresponding genes in seven types of AS. **(B)** Numbers and percentages of prognosis- associated AS events and corresponding genes. **(C)** UpSet plots showing the intersection of seven types of AS events. **(D)** UpSet plots showing the intersection of survival-associated AS events.

After processing and integrating AS data from TCGA SpliceSeq, and gene expression and clinical data from TCGA-PAAD dataset, and a total of 173 patients were included in the analysis. In total, 45,313 AS events in 10,624 mRNAs were observed in PC, indicating that AS events are common in the development of PC. Specifically, there were 3,657 AA events in 2,595 genes, 3,118 AD events in 2,211 genes, 9,325 AP events in 3,725 genes, 8,733 AT events in 3,817 genes, 17,402 ES events in 6,751 genes, 205 ME events in 203 genes, and 2,873 RI events in 1,923 genes found after preliminary analysis (Figure 1A). An UpSet graph was generated to analyze the intersection among seven types of AS and to display the distribution of spliced genes in different splicing types (Figure 1C). One gene may have multiple types of splicing events, and ES was found to be the most predominant type (38%). On the contrary, there were still 1,790 genes that spliced once in one of the seven patterns.

### Identification of Prognostic AS Events

Then, a univariate Cox analysis on AS events was conducted to exam the prognostic significance of AS events in patients with PC. There were 1,565 AS events in 1,223 genes associated with the OS of patients with PC. To be specific, 155 AA events in 150 genes, 229 AD events in 126 genes, 290 AP events in 206 genes, 235 AT events in 165 genes, 647 ES events in 553 genes, 5 ME events in 5 genes, and 114 RI events in 112 genes were identified as prognostic AS events (Figure 1B). Moreover, one gene could have two or more AS events that were significantly associated with survival of patients with PC. Exon skip was still the leading prognostic AS type, and that a gene could have up to three prognostic events (Figure 1D).

### Construction of Prognostic Signatures for Patients With PC

**FIGURE 2:**
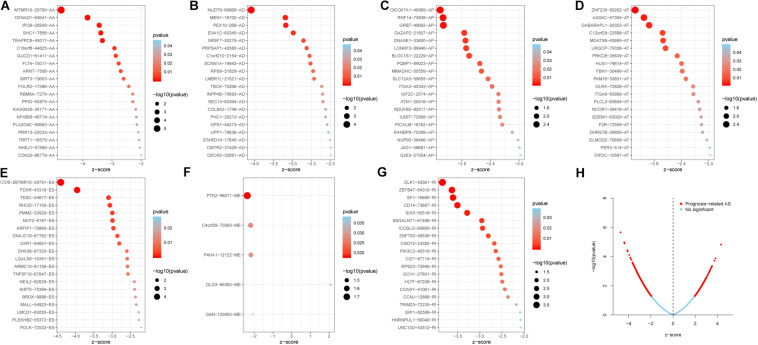
The top 20 most significant AS events in PC. **(A)** alternate acceptor, **(B)** alternate donor sites, **(C)** alternate promoters, **(D)** alternate terminators, **(E)** exon skips, **(F)** mutually exclusive exons, and **(G)** retained introns. **(H)** The distribution of AS events in PC cohort. The red dots represent AS events that are significantly associated with OS, while the blue dots are without correlation significance.

**FIGURE 3:**
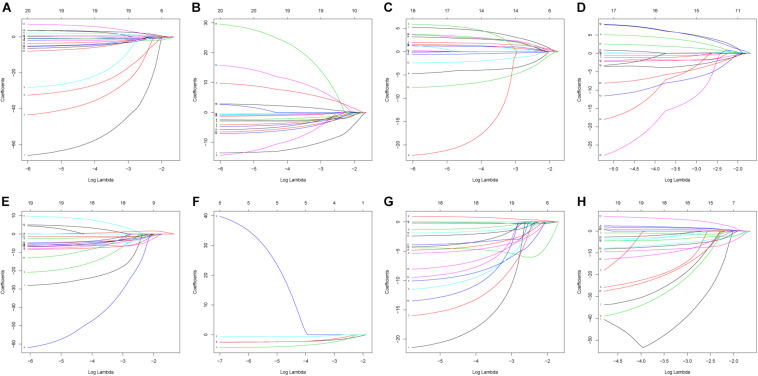
Construction of prognostic signatures based on LASSO COX analysis. **(A)** alternate acceptor, **(B)** alternate donor sites, **(C)** alternate promoters, **(D)** alternate terminators, **(E)** exon skips, **(F)** mutually exclusive exons, **(G)** retained introns, and **(H)** comprehensive signature.

**FIGURE 4:**
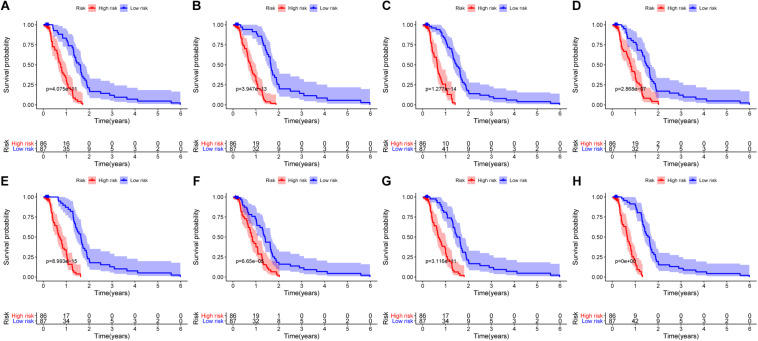
Kaplan–Meier curves of high risk (red) and low risk (blue) PC patients according to eight prognostic signatures. **(A)** alternate acceptor, **(B)** alternate donor sites, **(C)** alternate promoters, **(D)** alternate terminators, **(E)** exon skips, **(F)** mutually exclusive exons, **(G)** retained introns, and **(H)** comprehensive signature.

**FIGURE 5:**
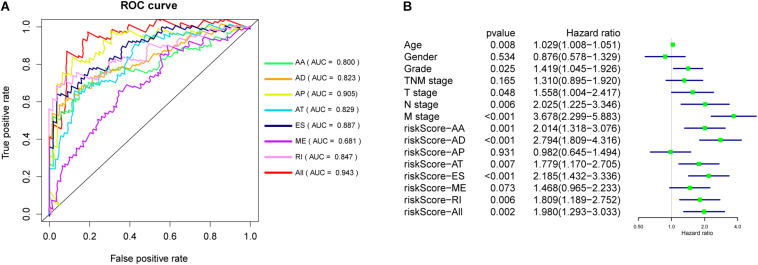
**(A)** ROC curves of prognostic signatures for PC. **(B)** Univariate Cox regression analysis of clinical features and prognostic signatures.

**FIGURE 6:**
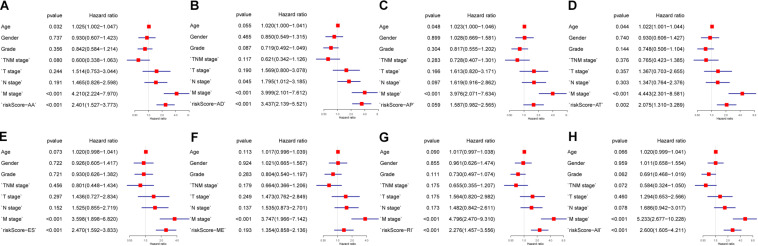
Multivariate analysis of clinicopathological features and eight prognostic signatures. **(A)** alternate acceptor, **(B)** alternate donor sites, **(C)** alternate promoters, **(D)** alternate terminators, **(E)** exon skips, **(F)** mutually exclusive exons, **(G)** retained introns, and **(H)** comprehensive signature.

The 20 most significant prognostic events of each of the seven AS types and the comprehensive view of AS events that significantly correlated with patient survival were demonstrated in Figure 2. Seven types of prognostic signatures were developed based on prognostic AS events using the LASSO Cox analysis (Figures 3A–G). Moreover, a comprehensive prognostic signature were generated by integrated analysis of all the seven types of AS events (abbreviated as “ALL”), which consist of 12 AS events (MTMR10-29790-AA, SLC20A2-83730-AP, NUDT9-69869-AD, FDXR-43316-ES, DLK1-93561-RI, OXNAD1-63641-AA, KANK1-85709-AP, NFKB2-12948-AA, DTNA-45096-AT, ACHE-81032-ES, ZBTB47-64310-RI and ELP3-83203-ES) (Figure 3H). The formulas of these prognostic signatures were summarized in Table 1. Furthermore, Kaplan–Meier analysis indicated that the eight prognostic signatures could effectively separate the survival curves of high-risk groups from those of the low-risk groups (Figures 4A–H). The efficacies of these eight prognostic signatures in prognosis prediction were validated by ROC curves, and the areas under the cure (AUC) of eight signatures were larger than 0.8, except for ME (AUC = 0.681), and the AUC of comprehensive signatures is 0.943 (Figure 5A). Univariate Cox regression analysis showed that six of eight signatures (AA, AD, AT, ES, RI, and ALL), as well as age, histologic grade, T stage, N stage, and M stage, had a high predictive value regarding the OS of patients with PC (Figure 5B). In addition, the above mentioned six signatures remained independent prognostic indicators for patients with PC in multivariate analyses after other clinicopathological characteristics were adjusted (Figure 6).

**TABLE 1:** Alternative splicing signatures associated with overall survival in patients with pancreatic cancer.

AS type	Formula	HR (95% CI)	AUC
AA	(MTMR10|29790|AA ×−71.23) + (OXNAD1|63641|AA ×−47.12) + (IPO8|20939|AA ×−28.79) + (RUFY2|11939|AA × 3.86) + (TRAPPC8|45017|AA ×−40.07) + (DLG4|38849|AA ×−6.22) + (DNAJC21|71747|AA × 8.29) + (POLM|79444|AA × 3.64) + (GMPPA|95766|AA ×−4.06) + (RNF121|17460|AA ×−5.29) + (GUCD1|61411|AA ×−7.21) + (PRRG2|50978|AA ×−8.78)	2.014 (1.318–3.076)	0.800
AD	(NUDT9|69869|AD ×−14.2) + (PPP1R7|58334|AD × 13.47) + (MFSD11|43685|AD ×−5.35) + (MEN1|16702|AD ×−17.4) + (FAM46A|76832|AD ×−4.54) + (PUM1|1443|AD ×−3.95) + (TXNDC17|38768|AD × 15) + (CLTCL1|61046|AD × 2.8) + (NRD1|2988|AD × 34.24) + (PDPN|713|AD ×−7.46) + (SPAG8|86314|AD ×−1.63) + + (ZFAND1|84310|AD ×−6.12) + (CENPW|77443|AD ×−5.31)	2.794 (1.809–4.316)	0.823
AP	(SLC20A2|83730|AP × 5.86) + (KANK1|85709|AP × 2.04) + (CLDN18|66949|AP × 6.57) + (TJP2|86531|AP × 2.38) + (RNF14|73838|AP ×−23.94) + (GRB7|40692|AP ×−6.58) + (ADPRHL1|26374|AP ×−2.88) + (MLLT3|85978|AP × 3.48) + (APTX|86073|AP × 2.87) + (TMEM243|80314|AP × 1.72) + (NFYB|24094|AP × 3.61)	0.982 (0.645–1.494)	0.905
AT	(DTNA|45096|AT × 7.65) + (ZNF695|10501|AT ×−0.71) + (ACYP2|53566|AT ×−4.88) + (KCNIP1|74491|AT ×−2.44) + (ZNF208|48800|AT × 5.4) + (DEPDC5|61896|AT ×−10.18) + (ZNF230|50262|AT ×−9.89) + (FHAD1|749|AT × 2.49) + (RNF32|82452|AT × 8.22) + (AADAC|67304|AT ×−18.98) + (CFLAR|56792|AT ×−7.79)	1.779 (1.170–2.705)	0.829
ES	(MTMR10|29791|ES ×−27.62) + (KIAA0922|70873|ES ×−4.61) + (INTS6|25947|ES ×−23.67) + (FDXR|43316|ES ×−64.23) + (ACHE|81032|ES ×−5.77) + (DDX19B|37348|ES ×−10.8) + (ELP3|83203|ES ×−6.91) + (HEXA|31549|ES ×−2.39) + (TUBD1|42826|ES ×−5.85) + (TMEM126B|18121|ES × 10.55) + (LRRC28|32678|ES ×−8.03) + (PLEKHB2|55371|ES ×−7.55) + (INTS7|9724|ES ×−12.46) + (KDM5C|89207|ES ×−5.7) + (PUM2|52774|ES × 6.56)	2.185 (1.432–3.336)	0.887
ME	(PTK2|98071|ME ×−2.64) + (C4orf29|70560|ME ×−2.5) + (P4HA1|12122|ME ×−4.8) + (DLG3|89383|ME × 43.86)	1.468 (0.965–2.233)	0.681
RI	(DLK1|93561|RI ×−20.93) + (ZBTB47|64310|RI ×−16.87) + (CD14|73687|RI ×−10.56) + (SIX5|50518|RI ×−13.35) + (MFSD11|43684|RI ×−6.28) + (C12orf73|24078|RI × 1.16) + (KRT15|40913|RI ×−1.92) + (B3GALNT1|67498|RI ×−18.48) + (POLR3H|62436|RI ×−5.12) + (EXOSC9|70501|RI ×−1.84) + (MPZ|8658|RI ×−8.1) + (ZNF169|86929|RI ×−6.98)	1.809 (1.189–2.752)	0.847
ALL	(MTMR10|29790|AA ×−68.75) + (SLC20A2|83730|AP × 3.51) + (NUDT9|69869|AD ×−11.11) + (FDXR|43316|ES ×−44.84) + (DLK1|93561|RI ×−27.79) + (OXNAD1|63641|AA ×−40.37) + (KANK1|85709|AP × 2.21) + (NFKB2|12948|AA ×−8.6) + (DTNA|45096|AT × 8.56) + (ACHE|81032|ES ×−10.5) + (ZBTB47|64310|RI ×−33.9) + (ELP3|83203|ES ×−16.26)	1.980 (1.293–3.033)	0.943

*AS, alternative splicing; HR, hazard ratio; AUC, area under curve; AA, alternate acceptor; AD, alternate donor sites; AP, alternate promoters; AT, alternate terminators; ES, exon skips, ME, mutually exclusive exons; RI, retained introns; ALL, all types.*

### Prognostic SF-AS Network

**FIGURE 7:**
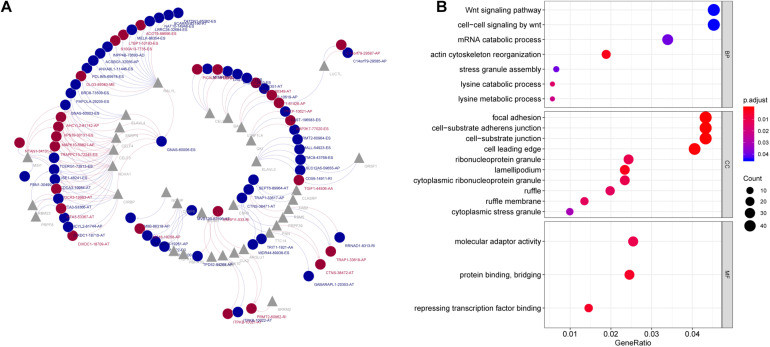
**(A)** Survival-associated SF-AS network in PC. Red/blue line represents positively/negative correlation; red/blue ellipse represents risk/protective AS events; gray triangle represents splicing factors. **(B)** Gene Ontology analysis of genes with survival-associated alternative splicing events. BP, biological process; CC, cellular component; MF, molecular function.

It is known to us that AS events are regulated by SFs (Sveen et al., 2016). Therefore, investigation of the SF–AS regulatory network is essential to study the mechanism underlying AS in PC. The results of correlation analysis suggested that there were 28 splicing factors were negatively correlated with 44 AS events, while 26 splicing factors positively correlated with 28 AS events. According to the correlation between SF and AS, an interaction network was constructed, which comprises 46 protective AS events (associated with good prognosis), 26 risk AS events (associated with poor prognosis) and 33 SFs (Figure 7A). Among the SF–AS network, splicing factors RALYL, NOVA1, and CELF3 were markedly associated with more than 12 AS events, and were considered as core SFs. On the other hand, protective AS event GNAS-60006-ES was positively associated with seven SFs, indicating that this event might play important roles in PC progression.

### GO Analysis

Gene ontology analysis were conducted on genes involved in prognostic AS events. The results suggested that these genes were associated with biological processes such as “Wnt signaling pathway,” “cell-cell signaling by Wnt,” and “mRNA catabolic process,” which were correlated to mRNA AS and pathogenesis of cancer (Figure 7B).

### Comparison With Previously Defined Molecular Clustering

**FIGURE 8:**
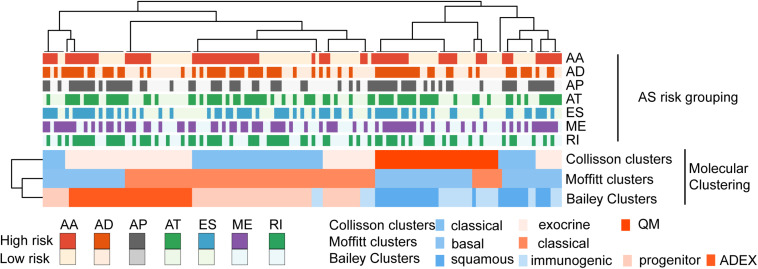
Comparison and correlation between these molecular clustering that have been previously defined and the risk grouping of AS events.

The categorization of PC patients into those with various AS events subgroup is for all intent a molecular subtyping approach. Gene expression studies have identified subtypes of PC with prognostic and biological relevance (Collisson et al., 2011; Moffitt et al., 2015; Bailey et al., 2016). A heatmap is generated to demonstrate the comparison and correlation between these molecular clustering that have been previously defined and the risk grouping of AS events identified in our study (Figure 8).

## Discussion

Previous literature reported that one gene can have multiple types of AS, different AS of the same gene may generate multiple mRNA and protein isoforms, which exert different biological effects. The number of AS events far exceeds the number of genes, which gave us a wider research prospective. The roles of prognosis prediction of gene expression signature have been well studied in the recent years. Although AS event signature do not have obvious advantage over the gene expression signature, we thought that investigation of prognostic role of AS deepens our understanding of posttranscriptional regulatory patterns.

Zhang et al. (2019) studied the AS events in 31 human cancer by integrally analyzing clinical data and splicing data from TCGA and SpliceSeq databases. In Zhang’s study, more attention was paid to the comparison and correlation of AS events among different cancer types. The author first provided profiles of AS events in 31 human cancers, and analyzed different AS events in 12 cancers. They also identified survival-associated AS events and prognostic signatures for 31 cancers, but their results are general, they did not analyze each specific type of AS. In our study, we analyzed the clinical and prognostic relevance of AS events and constructed prognostic signatures of all seven specific types of AS. Besides, univariate and multivariate Cox analyses were used to show that the all the constructed AS signatures could serve as independent prognostic factors for PC.

A total of 45,313 AS events in 10,624 mRNAs were observed in our study, suggesting that AS events are common in PC. Cox analyses indicated that 1,565 AS events in 1,223 genes are related with the survival of patients with PC. Seven splicing prognostic signatures were constructed based on seven types of survival-associated AS events. Moreover, a comprehensive prognostic signature was generated by integrating all seven types of AS. In the comprehensive prognostic signature, genes such as NFKB2 (Zhang et al., 1994), DLK1 (Lleres et al., 2019), KANK1 (Kariri et al., 2019), play important roles in cancer biology. Zhang et al. (1994) found that the NFKB2 gene rearrangement detected in HUT78 cells leads to the production of abnormal NFKB2 proteins capable of altering the function of the NF-kappa B transcription system. The comprehensive signature could serve as an effective tool to predict the prognosis of PC for its AUC value has reached 0.943. In addition, the constructed SF-AS network demonstrated that RALYL, NOVA1, and CELF3 might act as core SFs because of they were closely correlation with multiple AS events. Villate et al. (2014) reported that NOVA1 is an important regulator of AS in pancreatic beta cells. Moreover, NOVA1 regulates hTERT splicing, and NOVA1 knockdown significantly altered cancer cell growth in vitro and in xenografts in non-small cell lung cancer (Ludlow et al., 2018). Cui et al. (2012) found that RALY reduced expression is associated with poor prognosis in clear cell renal cell carcinoma. Further functional annotation analysis confirmed that the genes of these AS events have great potential to exert a crucial role in PC progression.

In conclusion, we identified survival-associated AS events in PC by analyzing the AS data from TCGA-PAAD database. The prognostic AS signatures could serve as promising prognostic indicators for PC patients.

## Data Availability Statement

Alternative Splicing and clinical data obtained from TCGA database.

## Ethics Statement

This study was conducted following the TCGA publication guidelines, and approval from a local Ethics Committee were unnecessary.

## Author Contributions

LX conceived and designed the study and wrote the manuscript. JP and YD collected and analyze the data. HP reviewed and edited the manuscript. All authors contributed to the article and approved the submitted version.

## Conflict of Interest

The authors declare that the research was conducted in the absence of any commercial or financial relationships that could be construed as a potential conflict of interest.
